# Positive Allosteric Modulator of SERCA Pump NDC-1173 Exerts Beneficial Effects in Mouse Model of Alzheimer’s Disease

**DOI:** 10.3390/ijms241311057

**Published:** 2023-07-04

**Authors:** Russell Dahl, Amanda C. Moore, Caitlynn Knight, Colleen Mauger, Hua Zhang, Gary E. Schiltz, Wendy A. Koss, Ilya Bezprozvanny

**Affiliations:** 1 Neurodon, 9800 Connecticut Drive, Crown Point, IN 46307, USA; cmauger@neurodon.net; 2 Purdue Institute for Integrative Neuroscience, Purdue University, West Lafayette, IN 47907, USA; amaulden@purdue.edu (A.C.M.); wkoss@purdue.edu (W.A.K.); 3 Department of Physiology, UT Southwestern Medical Center at Dallas, Dallas, TX 75390, USA; caitlynn.knight@utsouthwestern.edu (C.K.); hua.zhang@utsouthwestern.edu (H.Z.); 4 Department of Chemistry, Northwestern University, Evanston, IL 60208, USA; gary-schiltz@northwestern.edu; 5 Department of Pharmacology, Northwestern University Feinberg School of Medicine, Chicago, IL 60611, USA; 6 Robert H. Lurie Comprehensive Cancer Center, Chicago, IL 60611, USA; 7 Laboratory of Molecular Neurodegeneration, Peter the Great St. Petersburg Polytechnic University, 194021 St. Petersburg, Russia

**Keywords:** SERCA, calcium, Alzheimer’s disease, drug development, behavior, ER stress, UPR

## Abstract

Alzheimer’s disease (AD) is an irreversible neurodegenerative disease that affects millions of people worldwide. AD does not have a cure and most drug development efforts in the AD field have been focused on targeting the amyloid pathway based on the “amyloid cascade hypothesis”. However, in addition to the amyloid pathway, substantial evidence also points to dysregulated neuronal calcium (Ca^2+^) signaling as one of the key pathogenic events in AD, and it has been proposed that pharmacological agents that stabilize neuronal Ca^2+^ signaling may act as disease-modifying agents in AD. In previous studies, we demonstrated that positive allosteric regulators (PAMs) of the Sarco/endoplasmic reticulum Ca^2+^ ATPase (SERCA) pump might act as such Ca^2+^ stabilizing agents. In the present study, we report the development of a novel SERCA PAM agent, compound NDC-1173. To test the effectiveness of this compound, we performed behavioral studies with the APP/PS1 transgenic AD mouse model. We also evaluated effects of this compound on expression of endoplasmic reticulum (ER) stress genes in the hippocampus of APP/PS1 mice. The results of this study support the hypothesis that the SERCA pump is a potential novel therapeutic drug target and that NDC-1173 is a promising lead molecule for developing disease-modifying agents in AD.

## 1. Introduction

Alzheimer’s disease (AD) is an irreversible neurodegenerative disease that is currently the leading cause for dementia worldwide [1]. Current treatment options are very limited and focus primarily on comfort and symptomatic relief rather than disease regression and the prevention of further neuron loss. As the prevalence of AD increases with the growing population, identification of new drug therapies that can target synaptic and neuronal loss in AD is imperative. However, so far these efforts have yielded minimal positive outcomes [2,3,4,5]. Drug development efforts in AD are primarily based on the “amyloid cascade hypothesis” [4,5], and some very limited progress has recently been achieved using Aβ-targeting monoclonal antibodies. Therefore, there is a very significant unmet need to develop additional therapies for AD that may provide clinical benefits to patients. One potentially promising parallel approach is to develop therapies that do not target amyloid but rather, act on other signaling pathways. If successful, such an approach can be used as a monotherapy or in conjunction with amyloid-targeting therapies to augment their effects.

One such pathway is neuronal calcium (Ca^2+^) signaling. Neuronal Ca^2+^ dysregulation is a well-known feature of AD [6,7,8,9]. “Calcium hypothesis of aging and AD” has been proposed to explain cellular mechanisms of neurodegeneration and cognitive decline in aging [10]. Inhibitors of Ca^2+^ channels and Ca^2+^-signaling proteins have been considered as potential AD therapeutics. But most Ca^2+^-signaling proteins such as voltage-gated Ca^2+^ channels, ryanodine receptors, or calcineurin (CaN) play critical physiological roles outside the nervous system, which leads to potential “on target” side effects for such drugs [2]. Another potentially interesting and relatively unexplored Ca^2+^-related target is the Sarco/endoplasmic reticulum calcium ATPase (SERCA) Ca^2+^ pump. An integral endoplasmic reticulum (ER) protein, the SERCA pump acts as a key regulator of cellular Ca^2+^ homeostasis by actively transporting Ca^2+^ ions back into the lumen of the ER and preserving low cytosolic calcium levels. Thus, stimulation of SERCA pump activity by allosteric activators may potentially help to control cytosolic Ca^2+^ levels in AD neurons, with minimal side effects in contrast to Ca^2+^ signaling inhibitors. Indeed, some encouraging results have been already obtained with this class of compounds in preclinical models of AD in our previous studies [11].

In the present study, we describe the identification and evaluation of a novel positive allosteric modulator of the SERCA pump—2-methoxy-3-methyl-*N*-(2-methylquinolin-8-yl)benzamide (NDC-1173) [12]. Potential neuroprotective effects of this compound were evaluated in behavioral studies using the APP/PS1 transgenic mouse model of AD [13]. As an additional readout, we also used quantitative analysis of changes in endoplasmic reticulum (ER) stress markers levels following the APP/PS1 mouse treatment with NDC-1173, as ER stress is closely linked with dysregulated neuronal Ca^2+^ signaling and AD pathology in general. The obtained results support the potential utility of NDC-1173 and similar classes of compounds for treatment of AD neurodegeneration. 

## 2. Results

### 2.1. SERCA PAMs Optimization

**Figure 1:**
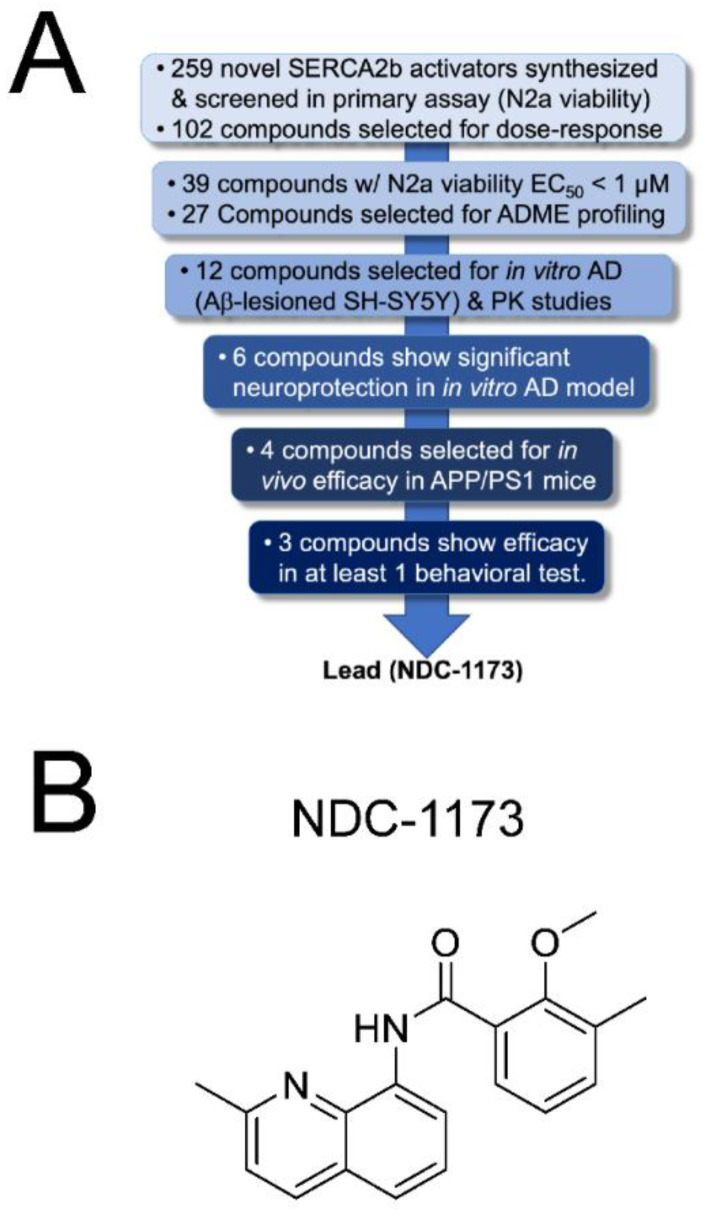
Development of NDC-1173. (**A**) A multi-stage testing funnel incorporating biological assays of increasing complexity was used to identify the lead SERCA2b activator. Using SBDD and iterative medicinal chemistry, 259 compounds were prepared and screened for their ability to protect N2a cells from ER stress-induced cell death. This produced 27 compounds with EC_50_ values less than 1 µM. Following assessment of ADME profiles, 12 compounds were chosen for mouse PK and assessment of neuroprotection in an in vitro AD assay consisting of SH-SY5Y neurons treated with a lethal amount of Aβ 1–42. Finally, four prioritized compounds were assessed in APP/PS1 mice with NDC-1173 showing the best overall profile in improvements of learning and memory. (**B**) Chemical structure of NDC-1173.

**Figure 2:**
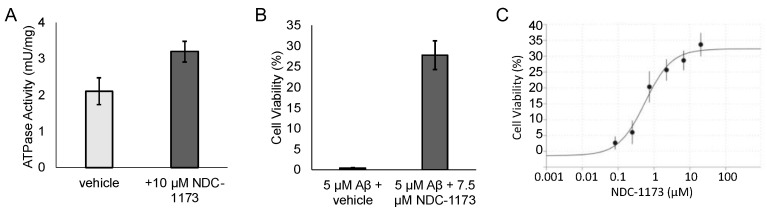
SERCA activation and neuroprotective effects of NDC-1173. (**A**) Calcium ATPase activity was assessed in mouse liver microsomes which contain only the endoplasmic reticulum subcellular fraction. ATPase activity was measured after 20 min incubation time in the presence of DMSO vehicle control or 10 µM NDC-1173. (**B**) Cell viability of SH-SY5Y cells in the presence of 5 µM Aβ 1–42 and vehicle or 7.5 µM NDC-1173. (**C**) Dose–response effect of NDC-1173 on viability of N2a cells in the presence of 15 µM thapsigargin. Mean ± SE (*n* ≥ 3).

Our previous research identified an aminoquinoline series of SERCA2b activators, exemplified by our early-lead CDN-1163 [11], which was developed via medicinal chemistry optimization of a series identified via HTS [14,15]. We subsequently confirmed, via Ca^2+^ imaging, that CDN-1163 restores normal Ca^2+^ homeostasis in APP/PS1 mice, which prompted us to undertake a lead optimization program to improve the potency and pharmaceutical properties of this series (Figure 1A). During the course of lead optimization, we synthesized over 250 novel SERCA2b activators using iterative SAR optimization assisted by structure-based drug design. As shown in Figure 1A, compounds were subjected to a rigorous testing funnel which included a primary ER stress viability screen in N2a cells to assess neuroprotection followed by dose–response potency evaluation, ADME, and finally, assessment in an in vitro model of AD consisting of SH-SY5Y neurons and Aβ. Compounds that met efficacy thresholds in the activity assays were then profiled in standard drug metabolism and pharmacokinetic (DMPK) assays to select compounds for in vivo assessment. At the conclusion of the in vitro selection process, we selected four candidate compounds—NUCC-0226446, NUCC-0226614, NUCC-0226646, and NDC-1173 for in vivo evaluation in the APP/PS1 transgenic mouse model of AD. The results of these in vivo studies are described in the following sections. Eventually our efforts resulted in the identification of the lead compound, NDC-1173 (Figure 1B), a drug-like, robust activator of SERCA [12]. The pharmacokinetic data for NDC-1173 are shown in Table 1. The SERCA activation data and key neuroprotection data in thapsigargin-treated N2a cells (primary screen) and Aβ 1–42-lesioned SH-SY5Y cells (secondary screen) are shown in Figure 2. Calcium ATPase activity (Figure 2A) was conducted in mouse liver microsomes which contain only the endoplasmic reticulum subcellular fraction, thus solely assessing SERCA activity. In addition, lead compounds, including NDC-1173, were assessed in a battery of other tests to determine selectivity for SERCA. This included effects on other channels and pumps including L-type Ca channel, Na/K-ATPase, and ryanodine receptor, and no significant functional effects were observed in any of these assessments.

### 2.2. Effects of SERCA PAMs in Object and Spatial Memory Tasks in an AD Mouse Model

**Figure 3:**
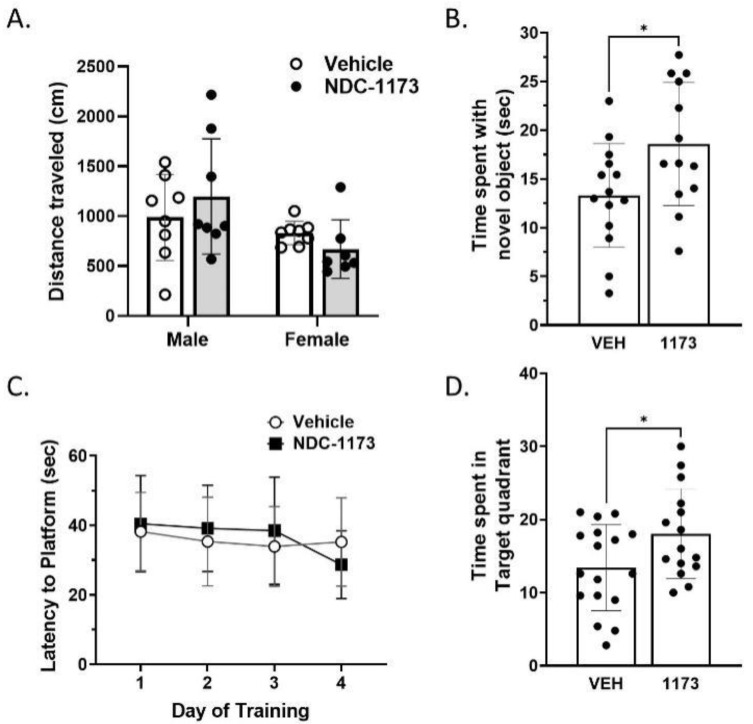
The effects of NDC-1173 on cognitive behavior of APP/PS1 mice. Administration of NDC-1173 did not (**A**) increase locomotor activity (VEH ♀ *n* = 8, ♂ *n* = 8; 1173 ♀ *n* = 7; ♂ *n* = 8) but a difference between the sexes was present. No cognitive behavior resulted in sex differences. In object recognition, NDC-1173 (**B**) increased object memory (VEH ♀ *n* = 8, ♂ *n* = 8; 1173 ♀ *n* = 6, ♂ *n* = 8). NDC-1173 (**C**) did not increase spatial learning across 4 days of the water maze (VEH ♀ *n* = 8, ♂ *n* = 8, 1173 ♀ *n* = 8, ♂ *n* = 9) compared to the APP/PS1 vehicle but did (**D**) increase spatial memory in the probe trial of the water maze. Statistical significance was determined by two- or three-way ANOVA (using treatment and sex factors) followed by two-tailed unpaired *t*-tests. Data represented as mean ± SD. * *p* < 0.05 compared to vehicle treated APP/PS1 mice.

To evaluate the effects of NUCC-0226446, NUCC-0226614, NUCC-0226646, and NDC-1173 on cognitive readouts in the AD mouse model, we treated APP/PS1 male and female mice with vehicle (10%DMSO/10%Tween80 in water) or SERCA PAMs (30 mg/kg) via oral gavage three times per week for 8 weeks, starting at 6 months of age. Open-field, object-recognition, and water maze assays were performed following treatment with 8-month-old mice. To test for locomotor activity changes in response to SERCA PAMs treatment in APP/PS1, we used the open-field test. The results obtained with NUCC-0226446, NUCC-0226614, NUCC-0226646 are shown in Supplementary Figure S1 and results with NDC-1173 are shown in Figure 3. For NDC-11173, a two-way ANOVA including sex and treatment as factors revealed no differences in distance traveled due to treatment in the open field test (Figure 3A). There was a significant sex difference in the open field (F_(1,27)_ = 16.75, *p* = 0.0237), but there was no sex by treatment interaction found.

Object recognition (OR) was used to examine the efficacy of NDC-1173 to reverse object memory deficits previously shown in APP/PS1 mice [16,17]. This task is dependent on the intact functioning of the hippocampus [18,19]. An improvement of memory in the group administered NDC-1173 was exhibited in the object recognition test (Figure 3B). An unpaired t-test revealed a significant increase in time spent with the novel object when compared to the vehicle (*p* = 0.0272). There were no significant sex differences. This result indicates that NDC-1173 can reverse deficits in object memory in APP/PS1. The Morris water maze (MWM) was performed to further examine the efficacy of NDC-1173 to reverse known deficits in spatial learning and memory [17]. The MWM has been a standard test of spatial learning and memory for decades and is dependent on hippocampal functioning [20]. No sex differences were apparent in any measure in our MWM experiments with APP/PS1 mice. The mice treated with NDC-1173 did not have better performance in spatial learning, as indicated by there being no differences in treatment between the treatment groups in the water-maze-training phase (Figure 3C). However, in the probe trial, mice treated with NDC-1173 spent more time in the quadrant where the escape platform was located 24 h before (*p* = 0.0364) (Figure 3D). This result supports that compound NDC-1173 improves spatial memory but not spatial working memory or learning in the APP/PS1 AD mouse model. The results obtained with NUCC-0226446, NUCC-0226614, NUCC-0226646 were largely consistent with results obtained with NDC-1173, but cognitive benefits were less pronounced with these compounds. No compound except NDC-1173 significantly improved the performance in both memory tasks (Supplementary Figure S1).

### 2.3. Effects of SERCA PAMs on ER-Stress Related Gene Expression in AD Mouse

**Figure 4:**
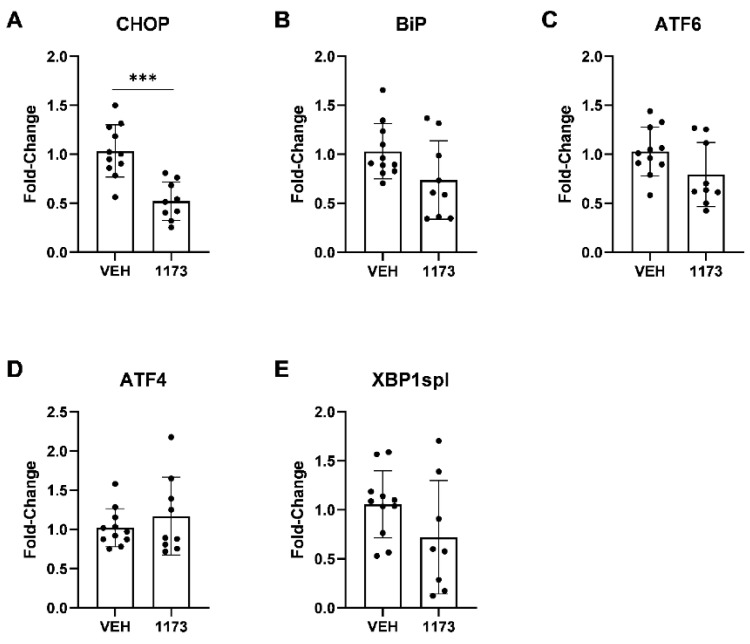
The effects of NDC-1173 on expression of ER-stress-related markers in APP/PS1 mice. (**A**) C/EBP homologous protein (CHOP/Ddit3), (**B**) binding immunoglobulin protein (BiP/Grp78), (**C**) activating transcription factor 6 (ATF6), (**D**) activating transcription factor 4 (ATF4), and (**E**) X-box binding protein 1 spliced variant (XBP1spl) were detected by real-time quantitative PCR and normalized to Cyclophilin. mRNA expression of ER-stress-related markers were reduced in APP/PS1 mice treated with NDN compound 1173 compared to the untreated vehicle group (VEH). Data expressed as mean ± SD (*n* = 9–11). Statistical significance determined by two-tailed unpaired *t*-tests. *** *p* < 0.001 compared to untreated APP/PS1 mice.

As previously established in Alzheimer’s disease (AD) research, ER stress is an apparent consequence of protein misfolding within AD pathophysiology. In response to this ER stress, cells activate a group of signaling pathways known as the unfolded protein response (UPR) that aims to restore ER function so the cell may survive. The expression of several UPR transcriptional factors and mediators, such as C/EBP homologous protein (CHOP), binding immunoglobulin protein (BiP or GRP78), activating transcription factor 6 (ATF6), activating transcription factor 4 (ATF4), and X-box binding protein 1 spliced variant (XBP1spl) have been reportedly increased in AD brains [21,22,23,24,25,26,27]. We examined the mRNA expression levels of these ER-stress markers in the hippocampus of APP/PS1 mice treated with NUCC-0226446, NUCC-0226614, NUCC-0226646, and NDC-1173 compounds compared to untreated APP/PS1 mice utilizing real-time quantitative PCR using primer sets indicated in Table 2. There was significant reduction (*p* = 0.0002) in the ER-stress marker, *Chop*, after treatment with NDC-1173 compared to untreated APP/PS1 mice (*n* = 9–11) (Figure 4, Table 3).

Other stress markers, excluding *Atf4*, were also reduced; however, given sample variability, the differences were not significant (Figure 4, Table 3). Treatment with compound NUCC-0226446 did not show any significant reductions in the expression of ER-stress-related markers (Table 3). The markers, *Atf6* and *Xbp1spl*, were significantly reduced after treatment with compounds NUCC-0226614 (*p* = 0.0022; *p* = 0.0328, respectively) or NUCC-0226646 (*p* = 0.0085; *p* = 0.0075, respectively) (Table 3). The *Bip* marker was significantly reduced after treatment with compound NUCC-0226646 (*p* = 0.0017) (Table 3). However, the reduction in these ER-stress markers were not as significant as the reduction in *Chop* after treatment with compound NDC-1173 (Table 3). CHOP, more frequently utilized, also acts as a more robust and reliable marker of ER-stress, as indicated in many previous studies [21,22,24,26,27,28,29,30]. Thus, we prioritized CHOP data over other markers in our analysis and concluded that NDC-1173 offered a robust benefit in reducing ER stress in the APP/PS1 AD model. Similar conclusions were reached based on analysis of ER stress marker expression via Western blotting using more limited set of samples.

**Table 2:** ER-stress-marker genes and primers. C/EBP homologous protein (CHOP/Ddit3), binding immunoglobulin protein (BiP/Grp78), activating transcription factor 6 (ATF6), activating transcription factor 4 (ATF4), and X-box binding protein 1 spliced variant (XBP1spl) with associated primer sequences used for qPCR experiments.

Marker	Primer Sequences	Source
**CHOP (Ddit3)**	FP 5′-GGAGGTCCTGTCCTCAGATGAA-3′ RP 5′-GCTCCTCTGTCAGCCAAGCTAG-3′	OriGene
**BiP (GRP78)**	FP 5′-GTTTGCTGAGGAAGACAAAAAGCTC-3′ RP 5′-CACTTCCATAGAGTTTGCTGATAATTG-3′	[22] [30]
**ATF6**	FP 5′-GTCCAAAGCGAAGAGCTGTCTG-3′ RP 5′-AGAGATGCCTCCTCTGATTGGC-3′	OriGene
**ATF4**	FP 5′-AACCTCATGGGTTCTCCAGCGA-3′ RP 5′-CTCCAACATCCAATCTGTCCCG-3′	OriGene
**XBP1spl**	FP 5′-GAGTCCGCAGCAGGTG-3′ RP 5′-GTGTCAGAGTC-CATGGGA-3′	[26]
**GAPDH**	FP 5′-CATCACTGCCACCCAGAAGACTG-3′ RP 5′-ATGCCAGTGAGCTTCCCGTTCAG-3′	OriGene
**Cyclophilin**	FP 5′-TGGAGAGCACCAAGACAGACA-3′ RP 5′-TGCCGGAGTCGACAATGAT-3′	Established previously in lab

**Table 3:** qPCR results. Fold-change of mRNA expression levels of ER stress-related markers were analyzed utilizing real-time qPCR, and normalized to Cyclophilin. Similar results were seen when normalized to the housekeeping gene GAPDH. Data expressed as mean ± SD (*n* = 7–11). Statistical significance determined by two-tailed unpaired *t*-tests. * *p* < 0.05, ** *p* < 0.01, *** *p* < 0.001 compared to untreated APP/PS1 mice.

ER-Stress Gene	NDC-1173	NUCC-0226446	NUCC-0226614	NUCC-0226646
**CHOP**	0.52 ± 0.195 ***	0.80 ± 0.280	0.86 ± 0.397	0.79 ± 0.223
**BiP**	0.74 ± 0.401	0.79 ± 0.378	0.82 ± 0.612	0.59 ± 0.163 **
**ATF6**	0.79 ± 0.327	0.80 ± 0.297	0.65 ± 0.228 **	0.71 ± 0.166 **
**ATF4**	1.17 ± 0.496	1.31 ± 0.494	1.01 ± 0.250	1.12 ± 0.452
**XBP1spl**	0.72 ± 0.577	0.88 ± 0.786	0.74 ± 0.238 *	0.61 ± 0.212 **

## 3. Discussion

### SERCA PAMs as Therapeutic Approach to AD

Alzheimer’s disease (AD) is a major health issue and leading cause of dementia worldwide [2]. The main drug development efforts in AD are based on the “amyloid cascade hypothesis”. According to this hypothesis, accumulation of the amyloid-β (Aβ) peptide in the brain is the main proximal cause of the disease [4,5]. During the last 20 years, targeting amyloid has been the main focus of drug development in the field of AD research [3,4,5], and some limited progress has been achieved recently, using an infusion of monoclonal antibodies against Aβ, such as Aducanumab (Biogen, Cambridge, MA, USA), Lecanemab (Esai/Biogen, Tokyo, Japan), and Donanemab (Eli Lilly, Indianapolis, IN, USA). In addition to the accumulation of amyloid, dysregulated neuronal Ca^2+^ signaling has long been recognized as one of the key pathogenic drivers of AD [6,7,8,9]. The “Calcium hypothesis of aging and AD” has been proposed to explain cellular mechanisms of neurodegeneration and cognitive decline in aging [10]. Dysregulated Ca^2+^ signaling in AD neurons results in overactivation of calcineurin [31], causes inhibition of autophagy [32], causes mitochondrial dysfunction [33,34], affects synaptic plasticity [35], induces ER stress [36], and causes deficits in memory function [31,34].

Because of well-established Ca^2+^ signaling abnormalities in AD, a number of Ca^2+^-related targets have previously been evaluated in AD animal models. However, no such targets have been identified that are clinically effective yet do not come with several adverse side effects or risks. Activity of the ryanodine receptor, an intracellular Ca^2+^ release channel that is highly expressed in the hippocampus and cortex, is reportedly increased in AD patients. The development of inhibitors for this receptor is difficult due to the importance of this receptor in other organs such as the heart. One such pharmacological inhibitor of this receptor, dantrolene, has had reported success in several AD cellular and animal models [37,38,39,40]. However, even though current studies show dantrolene to have neuroprotective effects, it has limitations due to its difficulty in crossing the blood–brain barrier (BBB) [41]. CaN is another target related to Ca^2+^ dysregulation that has to do with the downstream effects of elevated cytosolic Ca^2+^ concentrations mentioned above [31]. Hyperactivity of CaN ultimately leads to decreased autophagic flux [31,32]. Ideally, the inhibition of CaN would lead to increased autophagy and reduce the presence of plaque buildup in the brain. Indeed, significantly reduced incidence of AD in transplant patients chronically treated with CaN inhibitor FK-506 was revealed through retrospective analysis [42], strongly supporting the important role of CaN in AD pathogenesis. However, CaN has several different cellular functions throughout the body including roles in immune response, cell cycle control and apoptosis, and neuronal plasticity. Controlled brain-specific inhibition of CaN has yet to be discovered, and as of right now, generalized inhibition of this protein could lead to immunosuppression, hypertension, metabolic abnormalities, malignancies, and other side effects that are difficult to control for [31]. The cost far outweighs the benefit. Other such targets include neuronal-store-operated Ca^2+^ channels such as TRPC6 [43,44,45], but targeting these channels may result in detrimental effects due to their important role in kidney function.

An alternative approach to stabilizing neuronal Ca^2+^ signaling in AD neurons is not to inhibit Ca^2+^ channels (such as RyanR2) or Ca^2+^ effectors (such as CaN) but to facilitate Ca^2+^ clearance from cytoplasm to the ER. Such results can be achieved by pharmacological stimulation of the SERCA Ca^2+^ pump. The SERCA pump acts as a gatekeeper for ER Ca^2+^ homeostasis by pumping Ca^2+^ into the ER lumen, utilizing ATP hydrolysis, and subsequently keeping cytosolic Ca^2+^ concentrations low. Inhibition of this pump utilizing pharmacological inhibitors such as thapsigargin has reportedly yielded increases in ER stress due to Ca^2+^ dysregulation [46]. This establishes SERCA as a vital component of ER stress regulation. Importantly, several of the factors involved in the unfolded protein response (UPR) are reportedly been increased in AD brains [47]. We reasoned that allosteric activation of the SERCA pump would increase the amount of Ca^2+^ pumped back into the ER lumen, decreasing the amount of Ca^2+^ present in the cytosol and effectively reducing ER stress [48,49] and normalizing autophagic flux [32]. Reduced ER stress and restored autophagic flux is expected to reduce the presence of protein aggregates and relieve cognitive impairment in AD.

The development and evaluation of CDN-1163 SERCA PAM in our previous studies confirmed that such compounds can exert beneficial effects in APP/PS1 mice [11]. In the present study, we expand on these results and report the development of a novel and more effective SERCA PAM compound: NDC-1173 (Figure 1B). NDC-1173 outperformed the three other SERCA PAM compounds by enhancing memory in both object and spatial memory tasks in an AD mouse. The other compounds had less robust effects or only improved the performance of one task (Figure 3, Supplementary Figure S1). Analogous to our findings on behavior, qPCR quantification NDC-1173 demonstrated more substantial effects on ER stress markers when compared to the other three compounds (Figure 4, Table 3). Obtained results indicate that NDC-1173 is a promising lead molecule for developing disease-modifying agents in AD.

## 4. Materials and Methods

### 4.1. Methods Used in Selection of NDC-1173

#### 4.1.1. ER Stress Cell Survival Assay

Neuro 2A (N2a) cells were obtained from ATCC (cat number 89121404). ATCC provides analysis and authentication services for the cell lines they provide. The N2a cells were maintained in Dulbecco’s modified eagle medium (DMEM) with 10% fetal bovine serum (FSB), and 1% antibiotic-antimycotic (hereinafter referred to DMEM). The cells were recovered from cultures via trypsinization and seeded into 96-well plates at 2000 cells/well in 100 µL of DMEM. The plates were incubated overnight at 37 °C. Test compounds were prepared from DMSO stocks by dilution in DMEM to achieve the desired concentrations. Dose–response curves were obtained by preparing 5 concentrations of the test compounds using 3-fold serial dilutions. After the assay plates were incubated for 2 h, thapsigargin (TG) from DMSO stock diluted into DMEM was dispensed into each well for a final concentration of about 15 µM TG. After the plates were incubated overnight (16 to 24 h), Alamar Blue reagent (10% of total volume) was added to wells and absorbance was measured to enable assessment of viability. EC_50_ values were calculated from the dose–response curves.

#### 4.1.2. Cell Viability in SH-SY5Y Cells upon Aβ 1–42 Lesion

The studies involving SH-SY5Y cells were conducted at QPS (www.qps.com, accessed on 1 May 2023; Delaware Technology Park, 3 Innovation Way, Newark, DE, USA), a contract research organization. QPS supplied the SH-SY5Y cell line and was responsible for their authentication. SH-SH5Y cells were kept in culture medium (DMEM medium, 10% FCS, 1% NEAA, 1% L-Glutamine 200 mM, 100 µg/mL Gentamycin) until 80–90% confluency. Cells were maintained at 37 °C, 95% humidity, and 5% CO_2_. On day 1, cells were seeded in culture medium on 96-well plates at a cell density of 2.5 × 10^4^ cells per well. After 48 h, cells were pre-treated with 7.5 µM compounds for 1 h and lesioned by addition of 5 µM Aβ and maintained at 37 °C, 95% humidity, and 5% CO_2_ for 48 h when cell viability was determined via MTT assay.

#### 4.1.3. Pharmacokinetics

Pharmacokinetics of NDC-1173 were assessed in CD-1 mice. The compound was formulated at 1 mg/mL in DMSO/Tween 80/water (10/10/80, *vol*/*vol*/*vol*) and dosed at 2 mg/kg intravenously (i.v.) or 10 mg/kg via oral gavage (P.O.) in triplicate. Blood was drawn at 0.25 h, 0.5 h, 1 h, 2 h, 4 h, 8 h, and 24 h into EDTA-containing tubes, and plasma was harvested via centrifugation. Plasma (10 μL) containing 50% acetonitrile in water (5 μL) was added to 200 µL of ACN containing an internal standard. The samples were vortexed for 30 s. After centrifugation at 4 °C and 4000 rpm for 15 min, the supernatant was diluted 3 times with water. Then, 20 µL of diluted supernatant was injected into the LC/MS/MS system for quantitative analysis. The samples were injected onto an Agilent ZORBAX XDB-Phenyl 5 μ column (50 × 2.10 mm). Mobile Phase A was water with 0.1% formic acid. Mobile Phase B was acetonitrile with 0.1% formic acid. Separation was achieved using a gradient of 70% A/30% B to 0% A/100% B over 2.10 min. A Shimadzu LCMS-8050 equipped with a turbo ion spray source was used for all analytical measurements. Data were fit using WinNonLin https://www.certara.com/software/phoenix-winnonlin/, accessed on 1 May 2023.

#### 4.1.4. Calcium ATPase Activity

ATPase assays in mouse liver microsomes were carried out using the ATPase Assay Kit (Colorimetric; Cat. no. ab234055; Abcam, Cambridge, UK) as per the manufacturer’s instructions. Briefly, microsomes and either DMSO control or 10 µM NDC-1173 were incubated in provided assay buffer containing ATPase substrate and developed at 25 °C for 20 min, and A_650_ was measured using an Accuris SmartReader 96 Microplate Reader.

### 4.2. Synthesis of NDC-1173

Oxalyl chloride (2M in DCM) (3.8 mL, 7.600 mmol) and DMF (38.5 mg, 0.527 mmol) at 0 °C were added dropwise to a stirred solution of 2-methoxy-3-methylbenzoic acid (884.6 mg, 5.323 mmol) in DCM (10 mL). The solution was stirred for 1 h at room temperature under nitrogen atmosphere. The resulting mixture was concentrated to produce 2-methoxy-3-methylbenzoyl chloride. Then, the 2-methoxy-3-methylbenzoyl chloride was dissolved in DCM (5 mL) and added dropwise to a stirred solution of 2-methylquinolin-8-amine (800.1 mg, 5.057 mmol) and TEA (1.1 g, 10.871 mmol) in DCM (10 mL) at 0 °C. The resulting mixture was stirred for 1 h at room temperature under nitrogen atmosphere. LCMS showed reaction was completed. The mixture was quenched with 50 mL of water and extracted with DCM (30 mL). The organic layer was dried over anhydrous sodium sulfate and concentrated. The residue was applied onto a silica gel column with ethyl acetate/petroleum ether (1:3) to a produce 2-methoxy-3-methyl-*N*-(2-methylquinolin-8-yl)benzamide (1.1925 g, 76%) as a white solid. LCMS (ESI): [M + H]^+^ = 307. ^1^H NMR (300 MHz, DMSO-*d_6_*) δ 12.25 (s, 1H), 8.90–8.87 (m, 1H), 8.33 (d, *J* = 8.4 Hz, 1H), 8.02–7.98 (m, 1H), 7.67–7.64 (m, 1H), 7.59–7.50 (m, 3H), 7.27 (t, *J* = 7.7 Hz, 1H), 3.97 (s, 3H), 2.78 (s, 3H), 2.08 (s, 3H).

### 4.3. Methods Used in Behavioral Studies with AD Mouse Model

#### 4.3.1. Subjects

Male and female APP/PS1 mice (Stock # 034829, B6C3-Tg (APPswe, PSEN1dE9) 85Dbo/Mmjax) [13] were obtained from the Mutant Mouse Resource & Research Center at the Jackson Laboratory at 8–10 weeks of age. Mice remained in the colony at Purdue University until 6 months of age. Mice were maintained on a 12/12 h light/dark cycle with ad libitum access to food and water. All female mice were housed in 3–4 per cage. Males were housed 1–3 per cage due to fighting after arrival at our facility. Drug administration began at 6 months of age and continued for 8 weeks. Behavioral measures started at 8 months of age and mice were euthanized at 10 months of age. All behavioral tasks were performed in the Purdue Animal Behavior Core. All work with animals described in this manuscript was approved by the Purdue Institutional Animal Care and Use Committee (Protocol 1909001948; approved 23 September 2022).

#### 4.3.2. Drug Administration

Vehicle or NDC 1173 was administered via oral gavage 3 times per week for 8 weeks at a dose of 30 mg/kg in a vehicle containing 10%DMSO/10%Tween80 in water at a 3 mg/mL concentration. The dose was chosen so that the drug would be on board while the first signs of amyloid plaques appear (6 mos) and before the time the disease had fully progressed [13]. We chose to administer the drug 3 times per week to cause minimal stress to the mouse. Using this regiment, no adverse effects or weight loss were observed throughout the entire 8 weeks of administration.

#### 4.3.3. Open Field

Open field behavior was measured in a 40 cm × 40 cm × 30 cm arena. Mice were placed in the novel arena and were free to explore the arena for 5 min. Ethovision XT video tracking software (Version 15) as used to measure the distance traveled. This test was performed to observe any locomotor effects of the treatment that may confound cognitive measures. Since the same arena was used for object recognition this test was also used as the first habituation period for the object recognition test.

#### 4.3.4. Object Recognition

The object recognition task (OR) was performed as previously described [50,51]. Before the start of behavioral testing, mice were handled for 3 d and then habituated to the testing arena for 5 min/d for 2 d. During both handling and habituation, mice were acclimated to objects by placing a Lego Duplo brick (6.3 × 3.1 × 2.3 cm) in their home cage. During habituation, mice moved about freely in the apparatus without objects present. Following habituation, mice were trained in OR. In training, mice were exposed to two identical objects placed 3 cm away from each wall in the northwest and northeast corners of the arena. A date stamp and a combination lock were used for objects. To ensure that all mice received the same amount of exposure to the objects, training trials were not ended until mice had accumulated 30 s of total object interaction. Ethovision XT video tracking software (Version 15.0) was used to assess the length of interaction with each object by using the software’s 3-point tracking. Interaction was determined by the length of time the mouse’s nose was within 1 cm of a tightly drawn zone around each object. After completion of the training session, mice were returned to their home cages and were tested for deficits 24 h later. The 24 h time delay was chosen because of previous work reporting that male and female C57Bl6 mice remember the objects after 24 h [51]. During testing, one training object was replaced with a novel object. For instance, if mice were trained with 2 date stamps, the novel object was the combination lock and vice versa. Which object was novel in testing was counterbalanced to group and sex. Time spent interacting with each object was then measured using Ethovision XT software (Version 15). Mice completed testing when a total of 30 s of total interaction (familiar and novel) was reached. If a mouse did not reach 30 s of total interaction in 20 min, the data from that mouse were excluded. Because each mouse was required to interact with the objects for 30 s and the amount of time spent with the familiar object was dependent on the time spent with the novel object, comparisons were made using the time spent with the novel object. More time spent with the novel object is indicative of intact memory.

#### 4.3.5. Water Maze

The water maze consisted of 3 phases pre-training, training, and probe trial. In both pre-training and training, mice performed 4 trials per day with a maximum of 60 s per trial. Mice were placed into the pool from the North, South, East, and West sides of the pool in a semi-random pattern with each mouse entering the pool from each direction every day. All phases were completed in a large pool 120 cm in diameter with water levels that were approximately 90 cm deep and a temperature of 23 ± 1 °C. The water was mixed with nontoxic white paint to make the pool opaque which prevented the mouse from seeing the hidden platform during the task. The escape platform was a circular platform that was clear Plexiglas and 8 cm in diameter. Large black and white patterned posters acted as spatial cues during the task. If the mouse did not find the platform in 60 s the mouse was led by the experimenter to the platform. Mice had to stay on the platform for 10 s before the experimenter removed the mouse from the platform, dried them off with a towel, and placed them in a separate cage under warming lights to prevent cold stress. All mice completed one trial and did not start the next trial until all other mice performed the task. This resulted in a 15 min rest period in between trials. Pre-training was performed to decrease the confound of stress in response to water as well as to teach mice that an escape platform is within the pool. In this phase, the platform was placed in the middle of the pool 1 cm above the water. The platform had bright green tape around the base so that the mice could easily identify it. Pre-training was performed for 1 d. The day after pre-training, training began. Training lasted 4 days with a total of 16 trials. During training, mice had to find the hidden platform that was submerged 1 cm underwater. Every day of training the platform was in the same place which was the Northwestern quadrant of the pool. Ethovision XT (Version 15) tracking software recorded the latency to the platform. Twenty-four hours after the last day of training the probe trial commenced. During the probe trial, the platform was not present within the pool and the search pattern of the mouse is measured. More time spent in the quadrant where the platform was previously placed is indicative of better memory.

### 4.4. qPCR Experiments for ER Stress Markers

At 10 months of age, mice were rapidly decapitated, and the entire hippocampus was dissected and snap-frozen using liquid nitrogen. Tissue was stored at −80 ° C until extraction. Samples were homogenized and total RNA was isolated from the hippocampus using TRIzol Reagent (Invitrogen, Waltham, MA, USA; Cat 1556026) following the manufacturer’s protocol. Total RNA concentration and purity was then measured using a NanoDrop spectrophotometer. DNase treatment, using DNase 1 (Roche, Basel, Switzerland) was then performed on all samples utilizing 1 ug of RNA. Synthesis of cDNA was then completed utilizing SuperScript II RT (Invitrogen Cat V02226). Real-time qPCR was then performed on a QuantStudio6 real-time PCR machine (Applied Biosystems, Waltham, MA, USA). PowerUp SYBR Green Master Mix (Applied Biosystems Cat A25742) was used along with the specific primers found in Table 2. Cyclophilin and GAPDH were utilized as internal loading controls. All genes were validated for specificity and linearity prior to experiments. Data were exported from the RT PCR machine into an excel worksheet. %CVs were calculated between triplicate well CT values to rule out technical outliers. CT values were normalized to Cyclophilin. Relative expression was then analyzed following the comparative CT method ($2^{-∆∆CT}$ method).

### 4.5. Statistics

All data were analyzed using the statistical program, GraphPad Prism 9.2. Single-measure behavior data were analyzed using 2-way ANOVAs with sex and treatment as factors. Training in the water maze was analyzed using a 3-way, repeated-measures ANOVA with time, treatment, and sex as factors. However, no sex differences were found in any measure except distance traveled in the open field, so male and female groups were combined to increase the power of the analyses of all cognitive assays and followed up with two-tailed unpaired t-tests or, for multiple measures, a two-way repeated measures ANOVA were performed. Data from subjects that were 2 standard deviations away from the mean were considered statistical outliers and were not analyzed. Because no sex differences were apparent in cognitive behavior testing, sexes were not separated in the analysis of qPCR experiments. These assays used two-tailed unpaired t-tests to determine statistical significance.

## Figures and Tables

**Table 1:** Key profiling data of NDC-1173. The brain and plasma PK parameters were determined after oral administration in mice at 10 mg/kg.

Properties	NDC-1173
Brain concentration at 2 h	2.5 μM
Brain/plasma ratio	2.8
Brain t_1/2_	1.1 h
Plasma t_1/2_	0.9 h
C_max_	1.25 µM
AUC	375 ng·h/mL
F%	25.6

## Data Availability

Not applicable.

## Supplementary Materials

The supporting information can be downloaded at: https://www.mdpi.com/article/10.3390/ijms241311057/s1.

## References

[1] Lopez O.L. (2011). The growing burden of Alzheimer’s disease. Am. J. Manag. Care.

[2] Bezprozvanny I. (2022). Alzheimer’s disease—Where do we go from here?. Biochem. Biophys. Res. Commun..

[3] Asher S., Priefer R. (2022). Alzheimer’s disease failed clinical trials. Life Sci..

[4] Karran E., De Strooper B. (2022). The amyloid hypothesis in Alzheimer disease: New insights from new therapeutics. Nat. Rev. Drug Discov..

[5] Haass C., Selkoe D. (2022). If amyloid drives Alzheimer disease, why have anti-amyloid therapies not yet slowed cognitive decline?. PLoS Biol..

[6] Bezprozvanny I., Mattson M.P. (2008). Neuronal calcium mishandling and the pathogenesis of Alzheimer’s disease. Trends Neurosci..

[7] Stutzmann G.E. (2007). The pathogenesis of Alzheimers disease is it a lifelong “calciumopathy”?. Neuroscientist.

[8] LaFerla F.M. (2002). Calcium dyshomeostasis and intracellular signalling in Alzheimer’s disease. Nat. Rev. Neurosci..

[9] Berridge M.J. (2009). Calcium hypothesis of Alzheimer’s disease. Pflug. Arch..

[10] Khachaturian Z.S. (1989). Calcium, membranes, aging, and Alzheimer’s disease. Introduction and overview. Ann. N. Y. Acad. Sci..

[11] Krajnak K., Dahl R. (2018). A new target for Alzheimer’s disease: A small molecule SERCA activator is neuroprotective in vitro and improves memory and cognition in APP/PS1 mice. Bioorg. Med. Chem. Lett..

[12] Dahl R. (2020). Quinolines That Modulate SERCA and Their Use for Treating Disease. US patent.

[13] Jankowsky J.L., Fadale D.J., Anderson J., Xu G.M., Gonzales V., Jenkins N.A., Copeland N.G., Lee M.K., Younkin L.H., Wagner S.L. (2004). Mutant presenilins specifically elevate the levels of the 42 residue beta-amyloid peptide in vivo: Evidence for augmentation of a 42-specific gamma secretase. Hum. Mol. Genet..

[14] Cornea R.L., Gruber S.J., Lockamy E.L., Muretta J.M., Jin D., Chen J., Dahl R., Bartfai T., Zsebo K.M., Gillispie G.D. (2013). High-throughput FRET assay yields allosteric SERCA activators. J. Biomol. Screen.

[15] Gruber S.J., Cornea R.L., Li J., Peterson K.C., Schaaf T.M., Gillispie G.D., Dahl R., Zsebo K.M., Robia S.L., Thomas D.D. (2014). Discovery of enzyme modulators via high-throughput time-resolved FRET in living cells. J. Biomol. Screen.

[16] Denver P., English A., McClean P.L. (2018). Inflammation, insulin signaling and cognitive function in aged APP/PS1 mice. Brain Behav. Immun..

[17] Webster S.J., Bachstetter A.D., Nelson P.T., Schmitt F.A., Van Eldik L.J. (2014). Using mice to model Alzheimer’s dementia: An overview of the clinical disease and the preclinical behavioral changes in 10 mouse models. Front. Genet..

[18] Baker K.B., Kim J.J. (2002). Effects of stress and hippocampal NMDA receptor antagonism on recognition memory in rats. Learn Mem..

[19] Cohen S.J., Munchow A.H., Rios L.M., Zhang G., Asgeirsdottir H.N., Stackman R.W. (2013). The rodent hippocampus is essential for nonspatial object memory. Curr. Biol..

[20] Clark R.E., Broadbent N.J., Squire L.R. (2007). The hippocampus and spatial memory: Findings with a novel modification of the water maze. J. Neurosci..

[21] Azfer A., Niu J., Rogers L.M., Adamski F.M., Kolattukudy P.E. (2006). Activation of endoplasmic reticulum stress response during the development of ischemic heart disease. Am. J. Physiol. Circ. Physiol..

[22] Jangra A., Dwivedi S., Sriram C.S., Gurjar S.S., Kwatra M., Sulakhiya K., Baruah C.C., Lahkar M. (2016). Honokiol abrogates chronic restraint stress-induced cognitive impairment and depressive-like behaviour by blocking endoplasmic reticulum stress in the hippocampus of mice. Eur. J. Pharmacol..

[23] Ricobaraza A., Cuadrado-Tejedor M., Marco S., Perez-Otano I., Garcia-Osta A. (2012). Phenylbutyrate rescues dendritic spine loss associated with memory deficits in a mouse model of Alzheimer disease. Hippocampus.

[24] Nijholt D.A., de Graaf T.R., van Haastert E.S., Oliveira A.O., Berkers C.R., Zwart R., Ovaa H., Baas F., Hoozemans J.J., Scheper W. (2011). Endoplasmic reticulum stress activates autophagy but not the proteasome in neuronal cells: Implications for Alzheimer’s disease. Cell Death Differ..

[25] Li F., Zhang Y., Lu X., Shi J., Gong Q. (2019). Icariin improves the cognitive function of APP/PS1 mice via suppressing endoplasmic reticulum stress. Life Sci..

[26] Zhang K., Kaufman R.J. (2008). Identification and characterization of endoplasmic reticulum stress-induced apoptosis in vivo. Methods Enzym..

[27] Chen K.Y., Chen Y.J., Cheng C.J., Jhan K.Y., Wang L.C. (2020). Excretory/secretory products of Angiostrongylus cantonensis fifth-stage larvae induce endoplasmic reticulum stress via the Sonic hedgehog pathway in mouse astrocytes. Parasit Vectors.

[28] Hu H., Tian M., Ding C., Yu S. (2018). The C/EBP Homologous Protein (CHOP) Transcription Factor Functions in Endoplasmic Reticulum Stress-Induced Apoptosis and Microbial Infection. Front. Immunol..

[29] Oyadomari S., Mori M. (2004). Roles of CHOP/GADD153 in endoplasmic reticulum stress. Cell Death Differ..

[30] Kudo T., Kanemoto S., Hara H., Morimoto N., Morihara T., Kimura R., Tabira T., Imaizumi K., Takeda M. (2008). A molecular chaperone inducer protects neurons from ER stress. Cell Death Differ..

[31] Reese L.C., Taglialatela G. (2011). A role for calcineurin in Alzheimer’s disease. Curr. Neuropharmacol..

[32] Zhang H., Knight C., Chen S.R.W., Bezprozvanny I. (2023). A Gating Mutation in Ryanodine Receptor Type 2 Rescues Phenotypes of Alzheimer’s Disease Mouse Models by Upregulating Neuronal Autophagy. J. Neurosci..

[33] Schon E.A., Area-Gomez E. (2010). Is Alzheimer’s disease a disorder of mitochondria-associated membranes?. J. Alzheimers Dis..

[34] Supnet C., Bezprozvanny I. (2010). Neuronal calcium signaling, mitochondrial dysfunction, and Alzheimer’s disease. J. Alzheimers Dis..

[35] Chakroborty S., Goussakov I., Miller M.B., Stutzmann G.E. (2009). Deviant ryanodine receptor-mediated calcium release resets synaptic homeostasis in presymptomatic 3xTg-AD mice. J. Neurosci..

[36] Krebs J., Agellon L.B., Michalak M. (2015). Ca(2+) homeostasis and endoplasmic reticulum (ER) stress: An integrated view of calcium signaling. Biochem. Biophys. Res. Commun..

[37] Chakroborty S., Briggs C., Miller M.B., Goussakov I., Schneider C., Kim J., Wicks J., Richardson J.C., Conklin V., Cameransi B.G. (2012). Stabilizing ER Ca2+ channel function as an early preventative strategy for Alzheimer’s disease. PLoS ONE.

[38] Lacampagne A., Liu X., Reiken S., Bussiere R., Meli A.C., Lauritzen I., Teich A.F., Zalk R., Saint N., Arancio O. (2017). Post-translational remodeling of ryanodine receptor induces calcium leak leading to Alzheimer’s disease-like pathologies and cognitive deficits. Acta Neuropathol..

[39] Oules B., Del Prete D., Greco B., Zhang X., Lauritzen I., Sevalle J., Moreno S., Paterlini-Brechot P., Trebak M., Checler F. (2012). Ryanodine receptor blockade reduces amyloid-beta load and memory impairments in Tg2576 mouse model of Alzheimer disease. J. Neurosci..

[40] Peng J., Liang G., Inan S., Wu Z., Joseph D.J., Meng Q., Peng Y., Eckenhoff M.F., Wei H. (2012). Dantrolene ameliorates cognitive decline and neuropathology in Alzheimer triple transgenic mice. Neurosci. Lett..

[41] Abou M.B., Sun L., Wei H. (2020). Approaches to Optimizing Dantrolene Neuroprotection for the Treatment of Alzheimer’s Disease. Curr. Alzheimer Res..

[42] Taglialatela G., Rastellini C., Cicalese L. (2015). Reduced Incidence of Dementia in Solid Organ Transplant Patients Treated with Calcineurin Inhibitors. J. Alzheimers Dis..

[43] Zhang H., Sun S., Wu L., Pchitskaya E., Zakharova O., Fon Tacer K., Bezprozvanny I. (2016). Store-Operated Calcium Channel Complex in Postsynaptic Spines: A New Therapeutic Target for Alzheimer’s Disease Treatment. J. Neurosci..

[44] Lu R., Wang J., Tao R., Wang J., Zhu T., Guo W., Sun Y., Li H., Gao Y., Zhang W. (2018). Reduced TRPC6 mRNA levels in the blood cells of patients with Alzheimer’s disease and mild cognitive impairment. Mol. Psychiatry.

[45] Popugaeva E., Vlasova O.L., Bezprozvanny I. (2015). Restoring calcium homeostasis to treat Alzheimer’s disease: A future perspective. Neurodegener. Dis. Manag..

[46] Lytton J., Westlin M., Hanley M.R. (1991). Thapsigargin inhibits the sarcoplasmic or endoplasmic reticulum Ca-ATPase family of calcium pumps. J. Biol. Chem..

[47] Scheper W., Hoozemans J.J. (2015). The unfolded protein response in neurodegenerative diseases: A neuropathological perspective. Acta Neuropathol..

[48] Kang S., Dahl R., Hsieh W., Shin A., Zsebo K.M., Buettner C., Hajjar R.J., Lebeche D. (2016). Small Molecular Allosteric Activator of the Sarco/Endoplasmic Reticulum Ca2+-ATPase (SERCA) Attenuates Diabetes and Metabolic Disorders. J. Biol. Chem..

[49] Park S.W., Zhou Y., Lee J., Lee J., Ozcan U. (2010). Sarco(endo)plasmic reticulum Ca2+-ATPase 2b is a major regulator of endoplasmic reticulum stress and glucose homeostasis in obesity. Proc. Natl. Acad. Sci. USA.

[50] Koss W.A., Haertel J.M., Philippi S.M., Frick K.M. (2018). Sex Differences in the Rapid Cell Signaling Mechanisms Underlying the Memory-Enhancing Effects of 17beta-Estradiol. eNeuro.

[51] Koss W.A., Frick K.M. (2019). Activation of androgen receptors protects intact male mice from memory impairments caused by aromatase inhibition. Horm. Behav..

