# Supplementary material for: Positive Allosteric Modulator of SERCA Pump NDC-1173 Exerts Beneficial Effects in Mouse Model of Alzheimer’s Disease
Source: Int J Mol Sci. 2023 Jul 4;24(13):11057. doi: 10.3390/ijms241311057 (PMC10341805; doi:10.3390/ijms241311057)
Supplement: Supplementary file 1 [file ijms-24-11057-s001.zip › ijms-2483563-supplementary.pdf]

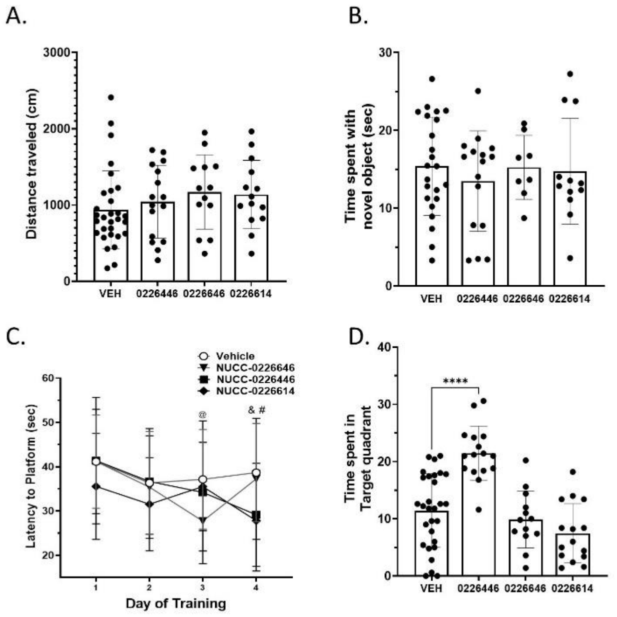

Supplementary Figure S1. The effects of SERCA PAMs on cognitive behavior of APP/PS1 mice. Administration of the compounds resulted in no differences in (A) locomotor activity as assessed in the open field (VEH ♀ *n* = 13, ♂ *n* = 16; 0226446 ♀ *n* = 9 ♂ *n* = 7; 0226614 ♀ *n* = 7; ♂ *n* = 7 and 0226646♀ *n* = 4; ♂ *n* = 4). (B) No differences were found in object memory when any of the compound-treated mice (0226446 ♀ *n* = 7 ♂ *n* = 8; 0226614 ♀ *n* = 7; ♂ *n* = 7; 0226646♀ *n* = 4; ♂ *n* = 4) were compared to vehicle-administered mice (VEH ♀ *n* = 11, ♂ n =12). In the water maze, (C) there were significant differences in specific days of water maze training. Mice treated with compound NUCC-0226646 (VEH ♀ *n* = 7, ♂ *n* = 6) performed better on day 3 and mice treated with NUCC-0226446 (VEH ♀ *n* = 9 , ♂ *n* = 9) or NUCC-0226614 (VEH ♀ *n* = 7, ♂ *n* = 7) had less of a deficit on day 4 when compared to the vehicle-treated mice (VEH ♀ *n* = 14, ♂ *n* = 15). In the (D) probe trial, only mice treated with compound NUCC-0226446 spent more time in the target quadrant. Data represented as mean ± SD. ****P<0.0001, @P<0.01 NUCC-0226646 vs. vehicle, &P<0.05 NUCC-0226446 vs. vehicle, #P<0.05 NUCC-0226614 vs. vehicle.
